# Seroprevalencia de *Trypanosoma cruzi* en niños de Veracruz, México: línea epidemiológica de base para un modelo de control fundamentado de la transmisión activa de la enfermedad de Chagas

**DOI:** 10.7705/biomedica.7126

**Published:** 2024-03-31

**Authors:** Ernesto Pérez-Sánchez, Raúl Montiel-Cruz, Eréndira Romero-Domínguez, Griselda Pascacio-Bermúdez, Arturo Báez-Hernández, Guadalupe Díaz-del Castillo Flores, Fabián Correa-Morales, Gonzalo Vázquez-Prokopec, Pablo ManriqueSaide, Azael Che-Mendoza, Gabriela Meneses-Ruiz, Irma López-Martínez, María Jesús Sánchez

**Affiliations:** 1 Servicios de Salud del Estado de Veracruz, Xalapa, México Servicios de Salud del Estado de Veracruz Servicios de Salud del Estado de Veracruz Xalapa Xalapa; 2 Centro Nacional de Programas Preventivos y Control de Enfermedades, Secretaría de Salud, Ciudad de México, México Centro Nacional de Programas Preventivos y Control de Enfermedades Centro Nacional de Programas Preventivos y Control de Enfermedades Ciudad de México Ciudad de México; 3 Department of Environmental Sciences, Emory University, Atlanta, GA, United States of America Emory University Emory University Atlanta Atlanta; 4 Unidad Colaborativa para Bioensayos Entomológicos, Campus de Ciencias Biológicas y Agropecuarias, Universidad Autónoma de Yucatán, Mérida, México Mérida Universidad Autónoma de Yucatán Mérida Universidad Autónoma de Yucatán; 5 Instituto de Diagnóstico y Referencia Epidemiológicos, Ciudad de México, México Ciudad de México Instituto de Diagnóstico y Referencia Epidemiológicos Ciudad de México Instituto de Diagnóstico y Referencia Epidemiológicos; 6 Organización Panamericana de la Salud, Ciudad de México, México Ciudad de México Organización Panamericana de la Salud Ciudad de México Organización Panamericana de la Salud

**Keywords:** Trypanosoma cruzi, enfermedad de Chagas, estudios seroepidemiológicos, niño, México, Trypanosoma cruzi, Chagas disease, seroepidemiologic studies, child, México

## Abstract

**Introducción.:**

En el 2021, la Secretaría de Salud de México y la Organización Panamericana de la Salud lanzaron una iniciativa para interrumpir la transmisión vectorial intradomiciliaria de *Trypanosoma cruzi*, fundamentada en la prevalencia de la enfermedad de Chagas en la población infantil. El estado mexicano de Veracruz fue el pionero de esta iniciativa.

**Objetivo.:**

Estimar la seroprevalencia de infección por *T. cruzi* en menores de 15 años de localidades rurales de Veracruz, México.

**Materiales y métodos.:**

Se identificaron ocho localidades prioritarias para la serología basal del municipio de Tempoal, Veracruz. Entre junio y agosto de 2017, se recolectaron muestras de sangre en papel filtro de 817 individuos para su tamizaje mediante un inmunoensayo enzimático de tercera generación. Los casos reactivos del tamizaje se confirmaron mediante pruebas de hemaglutinación indirecta, ensayo de inmunoabsorción ligado a enzimas e inmunofluorescencia indirecta en muestras de suero. Se calculó la seroprevalencia y su intervalo de confianza (IC) del 95 %.

**Resultados.:**

En las localidades de Citlaltépetl, Cornizuelo, Cruz de Palma y Rancho Nuevo se confirmaron casos de la enfermedad de Chagas en menores de 15 años con una seroprevalencia de 1,9 % (IC 95 % = 1,12-3,16).

**Conclusiones.:**

Los resultados indican que estas comunidades presentan transmisión reciente de *T. cruzi* y permiten establecer una línea epidemiológica de base para el diseño e implementación de un modelo dirigido a aquellas áreas geográficas con transmisión activa. Se espera que dicho modelo contribuya a la eliminación de la transmisión vectorial intradomiciliaria del tripanosomátido en México.

## Abstract (en)

**Introduction.:**

In 2021, the *Secretaría de Salud de México* and the Pan American Health Organization launched an initiative to interrupt intra-domiciliary vector transmission of *Trypanosoma cruzi* based on the prevalence of Chagas disease in children. The Mexican State of Veracruz was leading this initiative.

**Objective.:**

To estimate the seroprevalence of *T. cruzi* infection among children under 15 years of age from rural areas of Veracruz, México.

**Materials and methods.:**

We identified eight localities of high priority from the Municipality of Tempoal, Veracruz, for baseline serology. Blood samples were collected on filter paper from 817 individuals between June and August 2017, for screening with a third-generation enzyme immunoassay. Reactive cases were confirmed by indirect hemagglutination, enzyme-linked immunosorbent assay, and indirect immunofluorescence tests on peripheral blood serum samples. We calculated seroprevalence and 95% confidence intervals (CI).

**Results.:**

We confirmed Chagas disease cases in children under 15 years of age with a seroprevalence of 1,9% (95 % CI = 1,12-3,16) in the localities of Citlaltepetl, Cornizuelo, Cruz de Palma and Rancho Nuevo.

**Conclusions.:**

These results indicate recent transmission of T. cruzi in these communities and allow to establish an epidemiological baseline for the design and implementation of a model focused on geographical areas with active transmission to advance toward the elimination of intra-domiciliary vector transmission of this parasite in Mexico.

La enfermedad de Chagas, también llamada tripanosomiasis americana, es causada por el hemoflagelado *Trypanosoma cruzi* que es transmitido principalmente por vía vectorial a los humanos por las deyecciones de insectos triatominos (Reduviidae: Triatominae). La enfermedad de Chagas afecta a grupos vulnerables con alta marginación y representa un serio problema de salud, toda vez que puede reducir la calidad y la expectativa de vida de las personas ^1^.

En México, se estima que cerca de un millón de personas podrían encontrarse infectadas con *T. cruzi*^2^, aunque se reconoce que la carga de la enfermedad de Chagas está ampliamente subregistrada. La detección de casos de la enfermedad de Chagas en México se basa en una vigilancia pasiva, aunque en la última década se observó una tendencia creciente de casos por la disponibilidad de medicamentos y mejoras en el diagnóstico y registro ^3^^,^^4^. Desde 2017, la detección de casos incluye aquellos identificados por los bancos de sangre ^5^, caracterizados por una tasa de mortalidad baja y estable ^6^, y donde la mayor proporción de enfermos son crónicos. Por ejemplo, en el período 2017-2021 se registraron 3.250 casos de enfermedad de Chagas: 529 agudos y 2.721 crónicos ^7^.

Priorizar la búsqueda activa o intencional de los casos (sobre la búsqueda pasiva) para la detección temprana de Chagas en grupos en edad escolar (menores de 14 años) ha sido una demanda hecha por diversas iniciativas a los países de la región de las Américas desde hace más de 15 años ^8^^,^^9^. La vigilancia epidemiológica de estos grupos puede servir de centinela de la transmisión activa de la enfermedad ^10^^-^^12^, y puede controlarse mejor en este grupo de edad ya que tolera mucho mejor el tratamiento y presenta altos índices de curación (entre el 60 y el 100 %) ^13^. Si bien el “Programa de acción específico del programa de prevención y control de enfermedades transmitidas por vectores e intoxicación por veneno de artrópodos 20202024” de México ^14^ contempla la serología de menores de 15 años para la estratificación de riesgo de la enfermedad de Chagas, esta estrategia no ha sido adoptada por los programas estatales, por lo que la vigilancia entomológica continua siendo la principal actividad para la identificación de las áreas con transmisión y la aplicación de medidas de control.

Recientemente se estableció una iniciativa nacional para la implementación de un plan de acción para el abordaje de la enfermedad de Chagas en los municipios endémicos con la finalidad de lograr la interrupción de la transmisión vectorial con control sostenido domiciliario de *Triatoma dimidiata*^15^, el vector más ampliamente distribuido en México ^16^.

Para alcanzar el objetivo fijado en este plan de acción se implementarán las siguientes actividades:

- identificación de áreas (municipios y localidades) prioritarias para intervenir,
- establecimiento de la seroprevalencia base mediante la toma de muestras para serología en niños menores de 15 años y mujeres embarazadas,
- levantamiento de índices entomológicos antes del rociado de viviendas con insecticidas (índices de infestación intradomiciliaria y peridomiciliaria e índices de colonización),
- intervención de control vectorial: rociado integrado de viviendas con insecticidas de acción residual,
- evaluaciones posteriores al rociado: entomológicas y serológicas (al menos, 90 días después del rociado) y
- monitoreo de la sensibilidad a insecticidas.

Aquí se reportan las primeras dos actividades de este plan: la identificación de municipios y localidades prioritarias y el establecimiento de la seroprevalencia base (prevalencia de anticuerpos contra *T. cruzi*) mediante un estudio epidemiológico transversal en menores de 15 años de localidades rurales del estado mexicano de Veracruz en el 2017. Este estudio fue pionero de la “Estrategia de intervención nacional para la interrupción de la transmisión vectorial intradomiciliaria de la enfermedad de Chagas en México”.

## Materiales y métodos

### Identificación de áreas prioritarias (municipios y localidades)

**Figura 1:**
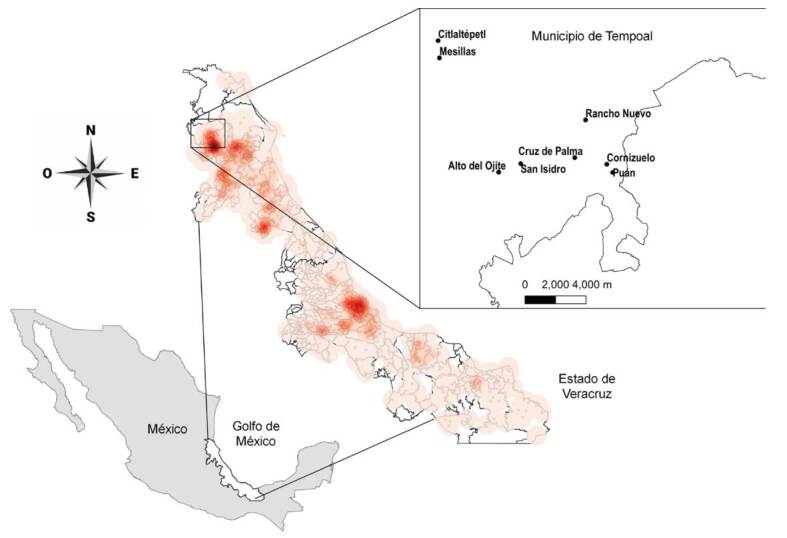
Distribución de los casos históricos acumulados de la enfermedad de Chagas (1990- 2016) y sitios de estudio. Mapa del estado mexicano de Veracruz que muestra las áreas de concentración de las localidades que reportan el mayor número de casos (mapa de calor basado en la estimación de densidad de Kernel). Se identifican varios focos importantes de transmisión. El recuadro (mapa superior derecho) muestra las ocho localidades del municipio de Tempoal identificadas como prioritarias y donde se realizó el estudio de seroprevalencia.

Se delimitó el universo de trabajo a las áreas geográficas de mayor riesgo y se identificaron en primera instancia los municipios prioritarios por intervenir (figura 1). Para esto, se partió de los datos históricos de casos de la enfermedad de Chagas -de 1990 al 2016- registrados en la Secretaría de Salud de Veracruz ^17^ y la presencia reportada del vector *T. dimidiata* del 2011 al 2017. De acuerdo con esta información se ponderó: a) con una puntuación de “1” si la localidad o municipio contaba con presencia de casos reportados (en menores de 0 a 5 años, entre 5 y 15 años, y mayores de 15 años) o “0” en caso contrario; b) número de casos totales de la enfermedad de Chagas que presenta, y c) “1” para la presencia reportada del vector (infectados o no infectados) en la localidad o “0” en caso contrario. Una vez seleccionados los municipios se procedió a seleccionar las localidades prioritarias usando los mismos criterios, pero incluyendo solamente localidades clasificadas como rurales con una población menor de 2.500 habitantes.

### Encuesta serológica basal en niños menores de 15 años

La detección serológica inicial (tamizaje) se realizó entre junio y agosto del 2017, previo censo de la población menor de 15 años. Se utilizaron lancetas retráctiles para la toma de muestra de sangre digital en papel filtro Whatman No. 1 (GE Healthcare UK Limited, Buckinghamshire, UK). Se usaron bolsas de plástico de cierre hermético, con desecante de gel de sílice en su interior para mantener las muestras secas y en buen estado durante su transporte y posterior envío al laboratorio.

La presencia de anticuerpos anti *T. cruzi* se determinó mediante un inmunoensayo enzimático de tercera generación (Accutrack Chagas Recombinante Microelisa test) en eluído de sangre seca en papel filtro. Los casos reactivos se confirmaron posteriormente mediante las pruebas de hemaglutinación indirecta, ensayo de inmunoabsorción ligado a enzimas (ELISA) e inmunofluorescencia indirecta (IFI) con muestras de suero de sangre periférica obtenida por punción venosa en el brazo (tomadas entre febrero y marzo del 2018) de acuerdo con la normatividad vigente en México ^3^^,^^18^.

Los casos estudiados se consideraron positivos, con presencia de anticuerpos contra *T. cruzi*, cuando sus muestras de suero resultaban reactivas, al menos, en dos de las tres pruebas practicadas ^3^.

Las muestras en papel filtro fueron embaladas y enviadas para su análisis al Instituto de Diagnóstico y Referencia Epidemiológicos en la Ciudad de México. Las pruebas confirmatorias con muestras de suero fueron realizadas en el Laboratorio Estatal de Salud Pública de Veracruz, México. En ambos casos el procesamiento de las muestras para el desarrollo de las pruebas diagnósticas se llevó a cabo en las primeras dos semanas después de haber sido tomadas.

Como parte de la normatividad que rige al programa de la enfermedad de Chagas, el diagnóstico serológico se extendió a todos los moradores de la vivienda (convivientes) si resultaba un caso positivo confirmado. A toda mujer embarazada detectada en el censo inicial se le hizo el diagnóstico serológico de la enfermedad de Chagas.

### Análisis de datos

Para el cálculo de los intervalos de confianza del 95 % (IC 95 %) y de la prueba exacta de Fisher para determinar si existían diferencias en las prevalencias por grupo de edad, se utilizó el *software* Stata 15™ (Stata Corp, College Station, TX).

### Consideraciones éticas

Todas las actividades fueron realizadas por el personal de la Secretaría de Salud de Veracruz, específicamente por el personal de Control de Vectores de la Jurisdicción Sanitaria de Pánuco, en el marco de las actividades de rutina realizadas como parte del programa de la enfermedad de Chagas en el estado ^14^.

Todos los casos con serologías positivas, tanto en los menores de 15 años como en las mujeres embarazadas y convivientes, se reportaron al Sistema Nacional de Vigilancia Epidemiológica (SINAVE) para su referencia y seguimiento, junto con el tratamiento etiológico correspondiente.

El estudio de la familia y la prescripción del tratamiento se realizó de acuerdo con los criterios y normativas mexicanas establecidos en la norma NOM-032-SSA2-2014 ^18^.

## Resultados

### Identificación de localidades prioritarias

Con los datos históricos de los casos de enfermedad de Chagas (1990-2016) de la Secretaría de Salud de Veracruz y la presencia reportada de *T. dimidiata* (2011-2017) se identificaron dos focos principales de transmisión que abarcan los municipios de Tempoal y Tantoyuca, al norte del estado (figura 1). Se seleccionó el municipio de Tempoal ya que, en los últimos cinco años, uno de cada cuatro casos (160/612) reportados en el estado pertenecía a este municipio, mientras que el municipio de Tantoyuca representó el 8,6 % (53/612) del total de casos de enfermedad de Chagas en Veracruz.

El municipio de Tempoal comprende 447 localidades, en su mayoría rurales, con una población total de 36.307 habitantes, distribuidos en 9.947 viviendas, y una población infantil (0-14 años) de 9.963 (27 %). La cabecera municipal Tempoal de Sánchez representa el 34 % (12.526) de la población total ^19^.

Se identificaron localidades prioritarias consideradas como focos de transmisión por el número de casos de enfermedad de Chagas. Para fines de la implementación de la “Estrategia de intervención nacional para la interrupción de la transmisión vectorial intradomiciliaria de la enfermedad de Chagas en México” -cuyas dos primeras acciones se reportan en este estudio- se seleccionaron las ocho localidades (figura 1) con mayor ponderación según el universo de trabajo establecido con los recursos disponibles del programa estatal de la enfermedad de Chagas.

### Prevalencia de infección por T. cruzi

Se tomaron 817 muestras de sangre en papel filtro a menores de 15 años de ocho localidades rurales del municipio de Tempoal, 178 (21,8 %) fueron de niños entre los 6 meses y menos de 5 años, y 639 (78,2 %) de niños entre 5 y menos de 15 años (cuadro 1).

**Cuadro 1:** Resultados de tamizaje de la enfermedad de Chagas a partir de muestras de sangre en papel filtro y pruebas confirmatorias (hemaglutinación directa, ELISA e inmunofluorescencia indirecta) con muestras de suero en población infantil de ocho localidades del municipio de Tempoal, Veracruz, México, 2017

Localidad/ Grupo de edad		Alto del Ojite	Citlaltépetl	Cornizuelo	Cruz de Palma	El Puan	Mesillas	Rancho Nuevo	San Isidro	Total
Infantes de 6 meses hasta menos de 5 años										
	Muestras	28	0	28	40	0	29	48	5	178
	Reactivas (tamizaje)	0	--	2	1	--	1	0	0	4
	Confirmadas (serología)	--	--	1	1	--	0	--	--	2
	Prevalencia (%)	--	--	3,6	2,5	--	--	--	--	1,1
	IC 95 %	--	--	0,1-18,3	0,1- 13,2	--	--	--	--	0,1- 3,9
Infantes de 5 hasta menos de 15 años										
	Muestras	127	1	95	163	7	95	146	5	639
	Reactivas (tamizaje)	0	1	7	5	0	1	6	0	20
	Confirmadas (serología)	--	1	4	5	--	0	4	--	14
	Prevalencia (%)	--	100	4,2	3,1	--	--	2,7	--	2,2
	IC 95 %	--	100-100	1,2-10,4	1,0- 7,0	--	--	0,7-6,9	--	1,2- 3,6
Total										
	Muestras	155	1	123	203	7	124	194	10	817
	Reactivas (tamizaje)	0	1	9	6	0	2	6	0	24
	Confirmadas (serología)	0	1	5	6	0	0	4	0	16
	Prevalencia (%)	--	100	4,1	2.9	--	--	2.1	--	1,9
	IC 95 %	--	100-100	1,3-9,2	1,1-6,3	--	--	0,6-5,2	--	1,1-3,2

De las 817 muestras, 24 (2,9 %) resultaron reactivas y de estas, 16 fueron confirmadas, es decir, la prevalencia de infección por *T. cruzi* fue del 1,9 % (IC 95 % = 1,12-3,16) en las ocho comunidades rurales del municipio de Tempoal, Veracruz (cuadro 1). No se encontraron diferencias estadísticamente significativas (F=0,54, p>0,05) entre las prevalencias de infantes entre los 6 meses y menos de 5 años (1,1 %; IC 95 % = 0,14-3,99) e infantes entre los 5 años y menos de 15 años (2,2 %; IC 95 % = 1,20-3,6).

Los resultados indicaron la presencia de menores de 15 años reactivos y confirmados (n=16) en las localidades de Citlaltépetl, Cornizuelo, Cruz de Palma y Rancho Nuevo.

Cincuenta y ocho convivientes fueron muestreados para serología confirmatoria, provenientes de las 13 viviendas de los infantes positivos. Se confirmó la infección por T. cruzi en el 13,8 % (8/58) de los convivientes, que corresponde al 53,8 % (7/13) de las viviendas con infantes positivos. La localidad de Rancho Nuevo presentó la mayor positividad de convivientes (3/11) y de viviendas (2/3), seguido de Cruz de Palma con 3 de 26 convivientes y 3 de 5 viviendas, y Cornizuelo con 2 de 17 y 2 de 4, respectivamente. No se encontraron convivientes positivos en Citlaltépetl.

Las viviendas en las que se encontraron convivientes positivos para *T. cruzi*, se pueden agrupar en dos tipos: i) viviendas con piso de tierra, techo de palma o de lámina de cartón o galvanizada, y paredes de otate con enjarre o adobe (lodo); y ii) viviendas con techos de losa y paredes de bloques de concreto sin revoque. En general, todas las viviendas de los casos confirmados presentaban grietas en su interior en paredes, acúmulos de leña o madera en la periferia o interior, con presencia de aves de corral y perros domésticos en libertad.

Finalmente, de las 82 muestras tomadas a mujeres embarazadas, solo una resultó reactiva y confirmada como positiva: provenía de una mujer de 30 años con 28 semanas de gestación de la localidad de Rancho Nuevo (1,2 %; IC 95 % = 0,03-6,6). La mujer convivía con uno de los menores que fue positivo para *T. cruzi.* El recién nacido fue valorado con serología al año con la cual se descartó enfermedad congénita de Chagas.

## Discusión

En México, la magnitud de la transmisión de la enfermedad de Chagas a nivel nacional se centra en dos estudios sobre seroprevalencia por infección de *T. cruzi* en población abierta. El primero reportó una prevalencia del 1,6 % (1.066/66.678) como parte de la Encuesta Nacional de Seroepidemiología realizada en México entre 1987 y 1989 ^20^. El segundo reportó una prevalencia del 1,5 % (996/64.969) en una encuesta realizada a donantes de sangre entre 1994 y 1996 ^21^. Un metaanálisis de los estudios publicados entre el 2006 y el 2017 estimó una seroprevalencia nacional del 3,38 % (IC 95 % = 2,59-4,16) en población abierta, del 1,51 % (IC 95 % = 0,772,25) en menores de 18 años y del 2,21 % (IC 95 % = 1,46-2,96) en mujeres embarazadas ^22^. En general, se estima que existen dos veces más casos de enfermedad de Chagas en las áreas rurales que en las urbanas ^20^. Aunque ninguno de estos estudios da detalle de la seroprevalencia en otros grupos de edad, se estima que esta aumenta con los años ^20^.

Los estudios específicos de infección por *T. cruzi* en población infantil (menores de 15 años) en México son escasos. En localidades rurales, la prevalencia reportada es del 0,4 % (3/685, grupo de edad ≤ 12 años) en Yucatán ^10^, del 6,2 % (22/356, 2-15 años) en el Estado de México ^23^, del 7,5 % (23/308, 0-14 años) en San Luis Potosí ^24^, del 9,9 % (13/131, 1-10 años) en Chiapas ^25^ y del 1,2 % (9/716, ≤ 6 años) en localidades rurales y urbanas de Colima ^26^.

La región norte de Veracruz históricamente representa algo más del 60 % de los casos de enfermedad de Chagas reportados por los servicios de salud del Estado ^17^. Para el grupo de menores de 15 años, los resultados del presente estudio epidemiológico arrojaron una prevalencia del 1,9 % (IC 95 % = 1,123,16). Estos resultados son similares a los reportes de localidades rurales de Veracruz entre el 2000 y el 2001, en los que se encontró una prevalencia del 0,91 % (IC 95 % = 0,85-0,94) en menores de 18 años. La región norte presentó los mayores niveles de prevalencia, entre el 2,5 y el 5,2 % ^11^.

En este trabajo, la prevalencia observada en mujeres embarazadas (1,2 %; IC 95 % = 0,03-6,6) es comparable con la encontrada en estudios previos en la zona norte de Veracruz que fue del 0,41 % (20/4.851) en Tuxpan (Ruiz A, Salazar PM, Rojas G, Guevara Y, Torres E, Gutiérrez M, *et al*. Seguimiento serológico de hijos de madres seropositivas a *Trypanosoma cruzi* en la jurisdicción sanitaria de Tuxpan, Veracruz, México. Memorias, XV Congreso Colombiano de Parasitología y Medicina Tropical. Biomédica. 2011;31(Sup.3):61) y del 3,5 % (3/85) en Poza Rica ^27^. Estos resultados aportan información relevante para dimensionar la intensidad de la transmisión de *T. cruzi* en la región norte del estado, que se encuentra entre las más altas reportadas en México para población infantil.

El norte de Veracruz presenta condiciones ambientales propicias para el establecimiento del vector combinadas con factores socioeconómicos y culturales que promueven la presencia de animales cerca de los hogares o dentro, la hacen una región importante para la transmisión de la enfermedad de Chagas. La calidad de la vivienda rural en esta zona es un factor importante en el riesgo de transmisión de *T. cruzi*^11^^,^^28^. Las viviendas de las localidades estudiadas tenían paredes hechas de madera, adobe o bloques de concreto que comúnmente contenían grietas o fisuras, las cuales sirven de refugio para los triatominos y facilitan su establecimiento, así como la presencia de corrales para animales de patio y animales domésticos en la vivienda, todos ellos factores de riesgo asociados a la seropositividad ^11^^,^^28^.

La vigilancia activa de la población infantil (menores de 15 años) como indicativo de la ocurrencia o no de transmisión vectorial activa, tiene implicaciones importantes en el control de la transmisión de *T. cruzi* y, por ende, en la incidencia de la enfermedad de Chagas. La experiencia en Honduras durante los años 90 introdujo un modelo que contempla la focalización de acciones según la estratificación del riesgo basada en la prevalencia en este grupo de edad ^29^. Este enfoque ha demostrado tener un impacto en la reducción de la infección por *T. cruzi*, en el tratamiento temprano de los casos agudos de la enfermedad de Chagas y la eventual interrupción de la transmisión ^29^.

**Figura 2:**
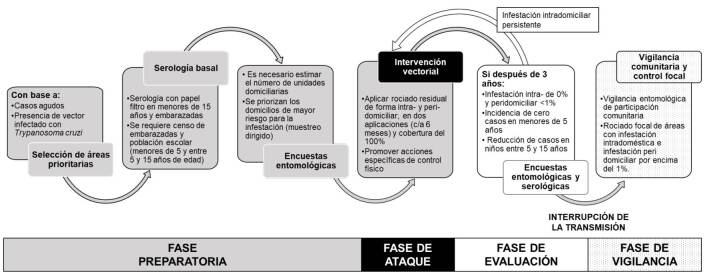
Estrategia para la interrupción de la transmisión vectorial intradomiciliaria de la enfermedad de Chagas en Veracruz, México

La información generada por el presente estudio ha sido utilizada por el “Programa estatal de prevención y control de la enfermedad de Chagas” para identificar localidades con transmisión activa de *T. cruzi* e implementar un plan de intervenciones dirigidas a interrumpir su transmisión vectorial intradomiciliaria (figura 2). En el plan se plasman las principales fases para alcanzar la interrupción de la transmisión vectorial por control sostenido domiciliario de *T. dimidiata* (vector con la mayor distribución en México) en un período mínimo de tres años: infestación vectorial intradomiciliaria del 0 % y peridomiciliaria menor del 1 %, incidencia de cero casos en menores de cinco años, reducción de casos en niños entre los 5 y los 15 años, e incidencia de cero casos agudos de enfermedad de Chagas; y, por último, el 100 % de cobertura de tamizaje para infección por *T. cruzi* en donantes de sangre ^15^.

El plan es el resultado de las actividades de cooperación técnica entre la Organización Panamericana de la Salud en México, el Centro Nacional de Programas Preventivos y Control de Enfermedades (CENAPRECE) de la Secretaría de Salud de México, los servicios de salud de Veracruz y la Universidad Autónoma de Yucatán. Este modelo de abordaje de la enfermedad de Chagas implementado en Veracruz está liderando los esfuerzos para avanzar hacia la eliminacion de la tranmision vectorial intradomiciliaria de *T. cruzi* en México y está incorporado dentro de los esfuerzos por fortalecer las acciones del programa nacional para el abordaje de la enfermedad de Chagas.
